# On-site IUD implementation in a student-run free clinic

**DOI:** 10.3389/frph.2026.1826766

**Published:** 2026-05-01

**Authors:** Ani Oganesyan, Joanna Lee, Kaitlyn Sweeney, Ellaina Villarreal, Kristine Jennings Burgess

**Affiliations:** 1 Chicago Medical School, Rosalind Franklin University of Medicine & Science, North Chicago, IL, United States; 2 College of Health Professions, Physician Assistant Program, Rosalind Franklin University of Medicine & Science, North Chicago, IL, United States

**Keywords:** community clinic, health equity, intrauterine device, long-acting reversible contraception, reproductive justice, student-run free clinic, uninsured

## Abstract

Uninsured populations face disproportionately high rates of unplanned pregnancy due to financial and structural barriers to effective contraception. While long-acting reversible contraception (LARC), such as intrauterine devices (IUDs), offers high efficacy for pregnancy prevention, implementation of IUDs within independent student-run free clinics (SRFCs) can be limited by a lack of hospital affiliation or government funding. This community case study details a sustainable model for integrating IUD services into an independent SRFC in Northern Illinois. By partnering with the humanitarian organization Direct Relief to secure donated devices and establishing a multidisciplinary workflow, the clinic successfully implemented a LARC program at a SRFC, demonstrating a sustainable approach to expanding reproductive health services for uninsured patients. The implementation process involved developing standardized clinical protocols, inventory management systems within the electronic health record, and interprofessional training for twenty-one student team members under faculty supervision. As of April 2025, the program is operational with a rolling waitlist and a projected capacity of one to two insertions per clinic session. This model demonstrates that independent SRFCs can expand reproductive health services for vulnerable populations through community partnerships and structured volunteer oversight, providing a replicable framework for other independent clinics to improve contraceptive access for the uninsured.

## Introduction

Access to contraception remains a fundamental component of equitable reproductive healthcare and comprehensive family planning, particularly for uninsured populations. Individuals without insurance experience higher rates of unintended pregnancy (1), and limited resources further exacerbate its consequences, underscoring the importance of access to effective contraception (2). LARC, including IUDs, are among the most effective forms of pregnancy prevention. However, they are associated with higher costs and limited public funding (3). Access to the full range of contraceptive options improves quality of care, optimizes health outcomes (4), and can improve patient autonomy and health literacy.

Student-run free clinics (SRFCs) play an important role in delivering care to uninsured communities. There are over 100 SRFCs that operate in the United States, typically serving uninsured populations (5) that comprised 8.2% or 27.2 million Americans of all ages in 2024 (6). Despite the need for specialty care, most SRFCs lack sufficient resources to sustain direct specialty care practices, and the ability of a given SRFC to provide such services is dependent on the extent to which that clinic is integrated into an umbrella academic institution (7). While some SRFCs have implemented LARC services through hospital partnerships or government-supported family planning grants, many do not offer such care (8). Sustainable, independent models remain underreported. As a result, there is limited guidance for SRFCs seeking to expand contraceptive services in resource-constrained environments.

While prior studies have described LARC implementation in student-run and community-based clinics, many of these models rely on hospital affiliation, institutional supply chains, or government-supported funding mechanisms. Hospital-affiliated SRFCs often benefit from institutional support, including provided supplies, equipment, medications, and ancillary services such as surgical procedures and imaging. For instance, the East Harlem Health Outreach Partnership Women's Health Clinic (EHHOP WHC) in New York is affiliated with the Mount Sinai Hospital Department of Obstetrics and Gynecology. Because the EHHOP WHC operates within the hospital gynecology clinic, supply costs are covered by the hospital, LARC devices are funded through a Family Planning Division grant, and patients can access no-cost imaging and surgical procedures via the hospital's social work and charity care programs (4). Less is known about how to implement and sustain these services within independent SRFCs operating without such infrastructure.

This community case study describes the development and implementation of IUD services within an independent SRFC operating without hospital affiliation or government funding. It outlines the processes of securing funding, establishing clinical protocols, training clinicians and healthcare student-volunteers, and integrating services into existing SRFC infrastructure. By detailing a replicable and sustainable implementation model, this work provides a practical framework for independent SRFCs seeking to expand reproductive health services in the absence of hospital affiliation or government funding and provides a practical framework for other SRFCs seeking to expand reproductive health services for uninsured populations.

### Context

To understand the development of this initiative, it is important to consider the clinical and community context in which care was delivered. The Interprofessional Community Clinic (ICC) is an independent, non-hospital-affiliated SRFC located in Lake County, Illinois, that provides longitudinal primary and specialty care to uninsured patients who face substantial financial, structural, and social barriers to accessing the traditional healthcare system. Patients served by the clinic primarily include individuals without health insurance, many of whom are underemployed, undocumented, or ineligible for public insurance programs. As a result, preventive reproductive health services such as contraception counseling and LARC are frequently delayed or inaccessible due to cost, transportation barriers, and fragmented referral pathways.

The ICC operates a dedicated Pelvic Health Clinic held monthly under the supervision of volunteer obstetrics and gynecology faculty. All faculty clinicians and student volunteers are covered under the university's malpractice plan. Specifically, students are provided with professional liability insurance while participating in registered coursework. The policy is issued by Columbia Casualty Company (CNA), with coverage limits of at least $1,000,000 per medical incident and an aggregate limit of $3,000,000 and is renewed annually to ensure continuous coverage.

Prior to implementation of IUD services, reproductive care within this clinic included contraception counseling, prescription of oral contraceptive pills, cervical cancer screening, and evaluation of gynecologic concerns. Although patients received counseling on the full spectrum of contraceptive options, IUD placement required referral to external organizations. Referral completion was inconsistent, as patients commonly encountered barriers including appointment wait times, transportation challenges, cost concerns, and unfamiliarity with external healthcare systems. While these observations were not systematically quantified, they represented a recurring and clinically significant gap in care within our patient population. Even when referrals were successfully completed, out-of-pocket costs and documentation requirements often limited access to LARC methods, which require procedural visits and device procurement. Consequently, patients seeking highly effective, long-acting contraception frequently lacked feasible options despite expressed interest. LARCs methods are particularly well-suited to our patient population, as they provide highly effective, long-acting contraception that does not require frequent follow-up or ongoing access to pharmacy services - barriers that are commonly encountered by uninsured patients. This further supported the decision to prioritize implementation despite the absence of formal baseline metrics.

SRFCs play an important role in addressing healthcare disparities by expanding access to preventive services for uninsured populations; however, most independent SRFCs do not offer procedural reproductive health services such as IUD insertion. The ICC operates out of the Rosalind Franklin University Health Clinics, which provides only clinic space and facilities. Supplies, equipment, and LARC devices must be independently obtained by students and faculty volunteers. This case study demonstrates the feasibility of providing reproductive services in an independent SRFC setting without reliance on hospital infrastructure. Notably, several SRFCs also operate in non-hospital community settings, including churches, homeless shelters, or through street outreach, where clearly defined pathways for reproductive services are similarly valuable (9). Common barriers include device cost, lack of institutional affiliation, limited procedural training opportunities, and uncertainty regarding workflow standardization and liability coverage. Unlike hospital-affiliated SRFCs that may rely on institutional funding or Family Planning Division grants, the ICC operates independently through volunteer faculty supervision and philanthropic support, necessitating alternative strategies for service expansion.

The initiative to implement IUD insertion emerged organically from both patient demand and clinician recognition of unmet contraceptive needs within the clinic population. Students and faculty identified LARC access as a critical gap in care, particularly given the clinic's emphasis on longitudinal relationships and continuity of care. Addressing this gap required developing an on-site IUD insertion service designed to operate within the existing structure and resources of a SRFC.

### Programmatic elements

Direct Relief is a non-profit, nongovernmental international organization dedicated to providing medical aid to regions impacted by poverty or emergency (10). The partnership between Direct Relief and the ICC was established via a formal application process, through which the ICC was recognized as a qualifying clinic serving the underserved and uninsured populations of Lake County, Illinois. This collaboration grants the clinic access to Direct Relief's medical catalog, facilitating the acquisition of Bayer® levonorgestrel-releasing IUDs (Mirena®, Kyleena®, and Skyla®).

To promote procedural consistency, the IUD insertion protocol was adapted from manufacturer-issued Bayer® clinical guidance and standardized for clinical use (11–13). This framework established uniform processes for counseling, informed consent, insertion technique, and post-procedure monitoring, ensuring reproducible, safety-focused care. The ICC's exclusion criteria for IUD insertion were consistent with the Bayer® clinical guidance excluding known or suspected pregnancy; uterine cavity distortion; current or prior progestin-sensitive malignancy; known or suspected uterine or cervical malignancy; liver disease; untreated acute cervicitis or vaginitis; unexplained uterine bleeding; current IUD *in situ*; acute pelvic inflammatory disease; postpartum endometritis or infected abortion within the past 3 months; and hypersensitivity to any component of Bayer® levonorgestrel-releasing IUDs (11–13).

To support the management of donated devices, a formal storage and inventory protocol was developed with approval from ICC pharmacy leadership. This protocol defined secure storage procedures, inventory documentation, and device tracking within the electronic health record (EHR), while clearly designating personnel responsibilities. For implementation, standardized IUD kits provided by Direct Relief served as the primary source of procedural supplies. Each procedure setup was supplemented with ICC-stocked materials, and the final procedure cart included sterile gloves, speculums, a sterile tenaculum, an antiseptic solution, sterile scissors, gauze, and local anesthetic supplies, including lidocaine and spinal needles.

To ensure ease and accuracy of documentation, the clinic utilized standardized dot phrases to record patient understanding, verbal consent, anesthesia details, and personnel present, including the provider, advanced student, and a scribe. During the visit, the patient received comprehensive counseling from the provider and advanced student regarding the available IUD option, as well as the risks, benefits, and what to expect during and after the insertion. Significant disparities in contraceptive counseling persist due to intersecting language and cultural barriers. Many patients enter clinical encounters with limited exposure to the full range of birth control methods, such as LARC. This baseline knowledge gap necessitates a more robust, culturally tailored approach to patient education to ensure that ‘informed consent’ is truly informed and that all patients have equitable access to the most effective forms of pregnancy prevention. At the ICC, certified interpreters are available as needed for the education process, consent process and procedure. Translators are evaluated and certified by trained, fluent faculty based on their level of proficiency in medical interpretation. If no in-person translators are available, the interpreter service Language Line is utilized. This is standard practice within the broader clinic and serves to improve patient access and care.

Prior to the procedure, the healthcare team was briefed by the clinic leads and the supervising clinician. The clinic leads provided everyone with the documentation expectations and protocol. Additionally, advanced students received a copy of the written protocol and the faculty clinician reviewed the IUD procedures and protocols with the advanced student prior to the patient encounter. The clinician then discussed expectations for the workflow of the patient visit with the entire interprofessional team.

Preparation of the necessary supplies and the IUD was performed by a clinical manager, who organized the sterile field and monitored the duration of the visit. All IUDs are inserted by trained faculty clinicians (physicians, physician assistants, or nurse midwives) who have complete the Bayer® IUD certification program. Advanced students first-assist clinicians during the procedure at the discretion of the provider. All ICC student volunteers complete require training and advanced student must have completed their Obstetrics and Gynecology rotations prior to taking on this role.

Patients are counseled on contraceptive options by both the faculty clinician and the advanced student. Once the decision to place a levonorgestrel-releasing IUD is decided, all patients are counseled on pre-medication pain management of ibuprofen 600 mg to 800 mg 30 min before IUD placement. Prior to the IUD insertion patients are also offered a paracervical block. In patients electing cervical anesthesia one to two mL of lidocaine are injected into the anterior lip of the cervix for tenaculum placement. Then, one to two mL of lidocaine are injected into the cervical os and as the cervix relaxes, the needles is advanced through the cervical os and the additional lidocaine is injected into the uterus for additional analgesia and hydrodilation.

Following IUD insertion, patients are advised to reach out to the clinic with concerns. Due to the monthly function of the clinic, if there was an emergent concern, patients are recommended to seek emergency care when necessary. The clinic does offer urgent appointments outside of monthly clinic dates on a case-by-case basis. In one instance an IUD causing pain was removed. This was not an IUD that was inserted by out clinic but rather an IUD inserted seven years prior. If IUD patients seek emergency care they are encouraged to reach out after emergency room visits for documentation purposes and to arrange follow-up care.

After IUD placement the scribe, advanced student, and clinician ensured proper documentation. The levonorgestrel-releasing IUD type, lot number, and expiration date were entered into the electronic health record and verified by the clinical team.

Following the initial procedure, a debriefing session was held to identify areas for quality improvement, specifically the implementation of dedicated procedure-specific consent forms and the distribution of patient education pamphlets to enhance health literacy and clinic efficiency. These improvements aim to streamline future insertions and reduce patient time in the office. Following the clinic's first IUD insertion, the patient returned two months later for a follow-up appointment, during which a string check was successfully completed, and she was re-counseled on potential changes in her menstrual cycle and the long-term effectiveness of her chosen device. The patient was also asked about her experience and areas in which the clinic could improve patient care.

## Discussion

This community case study describes the implementation of on-site IUD services within an independent, non-hospital-affiliated SRFC serving uninsured patients. Prior to this initiative, patients at the ICC could receive comprehensive counseling and short-acting contraception, including oral contraceptive pills, but individuals interested in LARC were required to seek care outside of the clinic. In practice, this structural separation functioned as a barrier. Even when patients expressed clear interest in an IUD, navigating external systems introduced predictable barriers, including cost, transportation challenges, and unfamiliarity with outside health systems. By integrating IUD insertion directly into the Pelvic Health Clinic, the ICC reduced fragmentation and aligned contraceptive care with an established, trusted source of longitudinal services. In doing so, the clinic moved beyond counseling alone and toward structural intervention.

Consistent with the aims of community-based reproductive health innovation, the program was designed with local context and feasibility in mind. The most significant enabling factor was a partnership with Direct Relief, which enabled device procurement at no cost to patients or the clinic. However, implementation required more than acquisition. In a volunteer-driven, intermittently staffed clinic, sustainability depended on standardized workflows, including clearly defined team roles, procedural cart organization, EHR-based lot tracking, and structured documentation through dot phrases. These operational elements ensured that high-quality contraceptive care relied on reproducible systems, reducing variability and supporting safety despite annual staffing changes.

An important insight from this project is that procedural reproductive health services can be embedded within community clinics through iterative refinement. A structured debrief after the first IUD insertion led to changes in consent processes, supply setup, and patient education materials. Rather than attempting to design a fully optimized system in advance, the clinic adopted a stepwise implementation model with real-time adjustments. This approach also further strengthens the clinic's relationship with its patients, taking into account their experiences and feedback to improve services and ensure long-term continuity of care. By implementing this stepwise approach, continuous improvements in patient care can be achieved.

Following the implementation of the IUD insertion and subsequent quality improvement meetings, we are continuously monitoring the protocol's clinical effectiveness. With IUDs now stocked in-house, patients receive direct counseling on the opportunity for same-day or scheduled placement. Current waitlists suggest interest from an additional five patients. We follow up with interested candidates monthly to manage scheduling and continue to collect monthly outcome data. We anticipate reaching a total of five patients by the end of the year with the goal of further expansion of the service in the following year.

The ICC's structure further strengthened implementation. Because reproductive care was established within a broader pelvic health clinic, IUD insertion was integrated into comprehensive counseling about menstrual changes, pain expectations, and follow-up. This model reflects a reproductive justice-oriented approach in which access to highly effective contraception is provided within a supportive, longitudinal care relationship rather than as a stand-alone procedural encounter. Implementing LARC services in a trusted setting may be particularly meaningful for uninsured and undocumented populations who experience healthcare exclusion and system mistrust.

The interprofessional structure of ICC, which includes medical, physician assistant/associate, physical therapy, pharmacy, and other health professional students, provided a collaborative environment conducive to developing standardized workflows and shared procedural responsibilities. Integration with pelvic floor physical therapy services also created an opportunity to support patients experiencing pelvic discomfort, anxiety, or post-procedural symptoms, allowing contraceptive care to be delivered within a broader model of comprehensive pelvic health. This initiative also highlights the dual clinical and educational impact of procedural integration in SRFCs dedicated to reproductive care. Students were involved in protocol development, inventory tracking, informed consent discussions, procedural implementation, and quality improvement conversations. In doing so, the project fostered trainee engagement with structural determinants of contraceptive access. For community clinics affiliated with health professions training programs, this represents an additional benefit: building future clinicians who are attentive to systems-level barriers in reproductive health care delivery, while also allowing students to participate in problem-solving initiatives.

From a broader perspective, this case contributes to the literature on expanding LARC access outside of hospital-affiliated or grant-funded environments. Many published models rely on institutional infrastructure or dedicated public funding streams, such as Title X family planning service grants. The ICC's experience as a non-profit organization suggests that strategic partnerships, standardized workflows, and iterative quality improvement can enable independent community clinics to incorporate procedural contraception services. While outcomes data are forthcoming, the establishment of durable infrastructure and a scalable operational model demonstrates feasibility.

**Figure 1:**
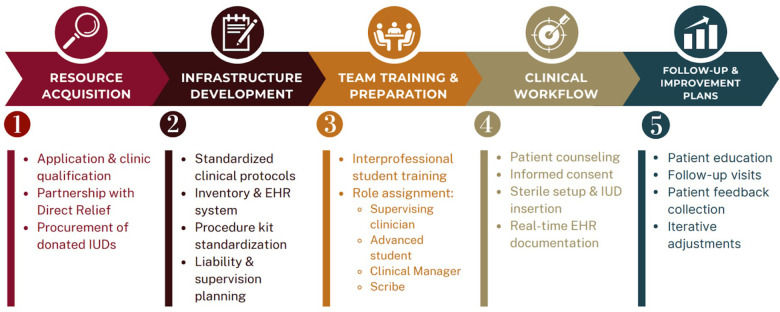
IUD Implementation in an Independent SRFC Workflow.

In summary, this community-based implementation illustrates how an independent, non-hospital-affiliated SRFC identified a local gap in contraceptive access and responded with a systems-focused intervention. By moving LARC provision from an external requirement to an integrated service within a trusted safety-net environment, the ICC advanced equitable access to highly effective contraception. This model may inform other community clinics seeking pragmatic, resource-conscious approaches to expanding reproductive health services and the provided figure details the suggested implementation workflow for clinics seeking to initiate a similar process (Figure 1).

### Limitations

Several limitations should be considered when analyzing this community case study.

First, this initiative was implemented within a single SRFC that already had a dedicated Pelvic Health Clinic and consistent volunteer faculty supervision. Clinics without specialty oversight, structured pelvic health appointments, or established longitudinal infrastructure may encounter additional barriers. As a result, the transferability of this model may depend on the presence of engaged supervising clinicians.

Second, a formal pre-implementation needs assessment was not conducted, as this initiative coincided with early clinic establishment and referral outcomes were not systematically tracked. The decision to pursue IUD services was based on recurring clinical encounters in which patients interested in LARC could only access methods such as oral contraceptive pills within the clinic or would need to seek LARC elsewhere. While this pattern was consistently observed by students and faculty, the absence of structured baseline data limits our ability to quantify unmet demand prior to service launch. A 2024 quality improvement effort found that four of 25 patients referred to the clinic had documented interest in contraceptive counseling, though specific interest in IUD placement was not documented. However, this likely underestimates the demand, as the assessment was limited to December 2023 to April 2024. Early outcome data remains limited. Five patients currently have documented interest in IUD placement. There have been no devices placed to date that have required removal. Waitlist data are limited due to changes in clinic tracking infrastructure. To support ongoing evaluation, a prospective quality improvement framework is being implemented to include standardized tracking of patient satisfaction assessment via structured surveys at follow-up (e.g., 6–12 months), and monitoring of clinical outcomes such as continuation rates at two months.

Third, outcome data remain early and limited. Although the infrastructure for insertion and documentation has been established, the number of completed insertions is small. The ICC has not yet collected longitudinal data on satisfaction, complications, or impact on contraceptive adherence compared to short-acting methods. Ongoing data collection will be necessary to evaluate sustained clinical and operational outcomes, and communication with patients is ongoing.

Fourth, procedural capacity is constrained by volunteer student and faculty availability. While device supply has been secured through a partnership with Direct Relief, the number of insertions per clinic session depends on supervising clinician availability and scheduling limitations within a monthly clinic model. Provider-student teams are able to see two patients, at 90 min per visit scheduled. Thus within these constraints, the maximum number of insertions per clinician is two, with the clinic maximum dependent on the number of trained providers present at any given clinic date – generally two to three provider-student teams per clinic. This may affect scalability and consistent access over time.

Finally, reliance on donated devices introduces potential uncertainty in long-term supply stability. Although the current partnership has enabled procurement at no cost to patients or the clinic, changes in donation availability or organizational policies could affect sustainability. Clinics adopting a similar model should consider contingency strategies for device sourcing. For the ICC, our ability to offer IUD services depends on the continued donation of levonorgestrel-releasing IUDs from Direct Relief. In the event that donations are no longer possible, several contingency plans can be employed. Nonprofit programs may establish partnerships with alternative nonprofit distributors such as Americares (14) or Medicines360 (15), which support contraceptive access for underserved populations. Additional approaches include using Patient Utilization Programs offered by manufacturers (e.g., Bayer® and Paragard®) (16, 17) to obtain devices at reduced or no cost. Finally, applying for grants from public health agencies, private foundations, or reproductive health organizations can provide supplemental funding streams to procure necessary supplies and maintain service continuity during periods of shortage.

These limitations underscore that, while feasible, integrating procedural LARC services into a community clinic requires ongoing evaluation and iterative quality improvement.

## Data Availability

The original contributions presented in the study are included in the article/Supplementary Material, further inquiries can be directed to the corresponding author.
